# Are There Differences between Methods Used for the Objective Estimation of Boar Sperm Concentration and Motility?

**DOI:** 10.3390/ani13101622

**Published:** 2023-05-12

**Authors:** Francisco Sevilla, Carles Soler, Ignacio Araya-Zúñiga, Vinicio Barquero, Eduardo R. S. Roldan, Anthony Valverde

**Affiliations:** 1 Costa Rica Institute of Technology, School of Agronomy, San Carlos Campus, Alajuela 223-21002, Costa Rica; 2 Costa Rica Institute of Technology, Doctorate in Natural Sciences for Development (DOCINADE), San Carlos Campus, Alajuela 223-21002, Costa Rica; 3 Department of Cellular Biology, Functional Biology and Physical Anthropology, Campus Burjassot, University of Valencia, C/Dr Moliner, 50, 46100 Burjassot, Spain; 4 Faculty of Agri-Food Sciences, Alfredo Volio Mata Experimental Station, University of Costa Rica, Cartago 11501-2060, Costa Rica; 5 Department of Biodiversity and Evolutionary Biology, National Museum of Natural Sciences, Spanish National Research Council (CSIC), 28006 Madrid, Spain

**Keywords:** boar, spermatozoa, reproduction, accuracy

## Abstract

**Simple Summary:**

Estimation of seminal quality can be carried out by examining different parameters. Such parameters directly influence the preparation of seminal doses used in artificial insemination in pigs. Sperm motility and concentration are relevant parameters in this context. The precision and accuracy of estimation of these parameters could thus contribute considerably to the productive performance of the pig industry. However, differences between systems could cause variations in estimates. These can be explained by the ability of the evaluator, type of system, or calibration, among others. In this study, we used iSperm^®^, ISAS^®^ v1, Open CASA v2, and the Accuread^®^ photometer system to compare assessments of motility and sperm concentration. Bayesian analyses were used, and differences were found between the four methods of concentration estimation. Additional analyses revealed differences in estimates of motility using these systems.

**Abstract:**

Artificial insemination in the swine industry, as in other species, demands adequate semen handling and accurate evaluation for the preparation of seminal doses. Sperm concentration and motility estimates are part of the semen evaluation process and are considered important for maximizing the yield of doses for insemination. In this study, methods were examined for their accuracy in the estimation of boar sperm concentration and motility. Assessments of sperm concentration were carried out using iSperm^®^, ISAS^®^ v1, Open CASA v2, and the Accuread^®^ photometer. Analyses of sperm motility were performed with iSperm^®^, ISAS^®^ v1, and Open CASA v2 systems. In this study, boar semen samples were collected from 10 healthy males from two genetic lines. There were no relevant differences between sire lines when sperm concentration was assessed. A Bayesian analysis was applied to the four methods used to assess sperm concentration to examine whether there are relevant differences between them. Results suggested differences in the four methods, with a probability of relevance (PR) of 0.86–1.00. The iSperm^®^ method revealed higher concentration values within the highest posterior density region at 95% confidence interval (HPD_95%_) = 167.0, 224.2 M/mL, whereas Open CASA v2 showed the lowest values, with HPD_95%_ = 99.3, 155.9 M/mL. The iSperm^®^ demonstrated higher reliability in measuring sperm concentration compared to other methods or devices within the given range of confidence. ANOVAs revealed relevant differences in the three methods of motility estimation. Overall, differences in boar sperm concentration and motility estimates were found using various methods, but further studies are needed for better characterization of these differences.

## 1. Introduction

The evaluation of seminal quality is very important for the success of artificial insemination in the swine industry [1]. Several factors may affect semen quality, such as season [2], individual differences [3], age [4], or sire line [5]. These could affect the fertility of inseminated sows when semen is used in artificial insemination [6]. Estimates of semen quality could be influenced by methods employed for evaluation. Accurate assessments are thus important, and efforts have been made to develop and use objective methods of semen analysis. The parameters regularly addressed in semen evaluation are known to vary depending on methodology, as in the case of sperm concentration [7,8,9], motility [10], viability [11], and morphological abnormalities [12,13].

The analysis of sperm concentration is an essential part of semen evaluation [2], and it is one of the most important parameters for the preparation of seminal doses [14]. Its estimation must be very accurate and be based on methods with high repeatability [15]. In the determination of concentration, there could be differences between systems [7,16,17]. The over- [18] or underestimation [14] could affect the selection of reproductive boars. Visual estimation is a traditional method used in pig farms [9], but this method is imprecise and has a lot of variation between technicians [17]. On the other hand, the Accuread^®^ (a photometer system) is carefully calibrated based on the accuracy of direct sperm counts, and it gives a highly repeatable estimation of sperm concentration [8]. CASA systems could improve accuracy, reliability and repeatability when used with the correct standardization and calibration [8].

Assessment of sperm motility may also show variation. This could depend on the analysis system used in the evaluation. The CASA systems have been used for many years [10], but their standardization has been difficult [19]. Also, the CASA systems could be expensive; therefore, other alternatives have been developed that allow these analyses to be carried out at a microscopic level and are more affordable for in-farm use [20,21].

The Open CASA v2 (a plugin of ImageJ software) is a free access software, and it provides access to seminal evaluations for those producers and researchers with lower resources. The system requires continuous validation and comparison with different analysis methodologies [22,23,24]. Open CASA v2 includes motility, morphology, concentration and vitality modules for seminal analysis [23]. The iSperm^®^ (Aidmics Biotechnology, Taipei, Taiwan) is a system that uses a smartphone or other hardware with the capacity to carry out a seminal analysis. This system does not require microscope lenses, lasers or other light objectives that can be very expensive, offering a convenient tool for multiple sectors [25]. The validation of these systems has been developed in animals such as dogs [26,27], horses [20,27,28], fish [22,29], and boar [30], but the number of studies is very limited. It is thus important to carry out additional research with these systems under several conditions for further characterization and to assess their accuracy and precision in comparative assessments.

The aim of this study was to evaluate the accuracy and the precision for assessments of boar sperm concentration using iSperm^®^ (Aidmics Biotechnology, Taipei, Taiwan), ISAS^®^ v1 (Proiser R + D, Paterna, Spain), Open CASA v2 (plugin of ImageJ software), and the Accuread^®^ photometer (IMV Technologies, L’Aigle, France). In addition, we examined the accuracy of estimates of total and progressive sperm motility using iSperm^®^, ISAS^®^ v1, and Open CASA v2 systems.

## 2. Materials and Methods

### 2.1. Ethics 

Studies were carried out in accordance with laws and regulations for conducting experiments on live animals in Costa Rica. Throughout the study, animals were handled carefully, avoiding any unnecessary stress. This study was performed following ethical principles and with the approval of the Committee of Centro de Investigación y Desarrollo en Agricultura Sostenible para el Trópico Húmedo at the Costa Rica Institute of Technology (CIDASTH-ITCR) according to Section 08/2020, article 1.0, DAGSC-100-2020. All the experiments were performed in accordance with relevant guidelines and regulations. The study was carried out in compliance with ARRIVE guidelines (https://arriveguidelines.org/; accessed on 9 February 2021).

### 2.2. Animals 

The experiments were conducted at a commercial pig farm (Agropecuaria Los Sagitarios S.A., Alajuela, Costa Rica) from May to November 2021 in the northwest of Costa Rica (Río Cuarto, 10°20′32″ N, 84°12′55″ W, Alajuela, Costa Rica). A total of 10 healthy and mature boars from two lines [Pietrain (*n* = 5); Duroc × Pietrain (*n* = 5)], of 17.4 ± 8.6 months of age at the beginning of the experiment, served as semen donors. During the study, the boars were individually housed in well-ventilated pens; the average temperature during the time of the study was 24.36 ± 2.79 °C. A standard breeder food mixture consisting of maize, soybean meal, a mix of minerals, and common salt was used [31]. Boars consumed an average of 2.5 kg/day and had water ad libitum.

### 2.3. Collection of Semen

The schedule for semen collection was regular morning collections, once a week, employing a “gloved hand” technique [32]. Semen was placed immediately in a water bath (37 °C). The sperm-rich fractions were used. They were diluted with a commercial extender (Zoosperm ND5; Import-Vet, Barcelona, Spain). The insemination doses were prepared in such a way that they contained 3.7 × 10^9^ spermatozoa. From each boar, an average of 4.80 ± 2.25 ejaculates were obtained (a total of 50 ejaculates). Each ejaculate was analyzed for subjective motility and sperm concentration using iSperm^®^, ISAS^®^ v1, Open CASA v2, and the Accuread^®^ photometer. Only ejaculates with >70% motile spermatozoa and >85% morphologically normal spermatozoa were employed in the study. Samples were transported to the laboratory under refrigeration (17 °C), conditions that were similar to those used in commercial practice. 

### 2.4. Assessment of Sperm Concentration and Motility with iSperm^®^

From each ejaculate, two subsamples of 250 µL were diluted 1:1 in ND5 extender and assessed using the iSperm^®^ system (Aidmics Biotechnology, Taipei, Taiwan). After thorough mixing of the diluted semen samples, 7.5 µL of semen was placed in the iSperm^®^ slide collector and pressed on a clean surface on the cover slide for two seconds. Once the sample was prepared, it was placed in the measurement equipment of the iSperm^®^ system, with the heater, which maintained the semen sample at 37 °C during the analysis. 

### 2.5. Assessment of Sperm Concentration and Motility with ISAS^®^ v1

The analysis of sperm concentration and motility was performed using a Makler^®^ chamber, which was pre-warmed to 37 °C. After semen samples were thoroughly mixed, a 2.7 µL aliquot was carefully pipetted and deposited in the center of the counting chamber. The cover glass was then gently placed on top of the counting chamber, taking extra care to avoid the formation of bubbles. Analyses were conducted using the CASA-Conc and CASA-Mot system ISAS^®^ v1 (Integrated Semen Analysis System, Proiser R + D, Paterna, Spain) fitted with a video camera (Proiser 782M, Proiser R + D), with a frame rate of 25 Hz and final resolution of 768 × 576 pixels. The camera was attached to a microscope UB203 (UOP/Proiser R + D) with a 1× eyepiece, a 10× magnification negative-phase contrast objective (AN 0.25), and an integrated heated stage maintained at 37.0 ± 0.5 °C. For concentrations >50 × 10^6^/mL, a dilution was required using the same extender; in general terms, a 1:2 dilution was needed. The CASA setting used was for a particle area between 10 and 80 μm^2^, and the connectivity was set at 11 μm. Total motility (%) refers to the percentage of spermatozoa that are actively moving, while progressive motility is characterized by the percentage of spermatozoa swimming forward quickly in a straight line. Progressively motile spermatozoa were those with straightness (STR, straightness index) of at least 45% and average path velocity (VAP) ≥ 25 µm·s^−1^, defined as the average velocity over the smoothed cell path. 

### 2.6. Assessment of Sperm Concentration and Motility with Open CASA v2

The ImageJ software and the Open CASA v2 Plugin [23] were installed, and the concentration and motility module were configured, using progressivity of 45% with respect to straightness (STR) with 25 frames per second. The minimum and maximum values for the parameters of the size of the cells were 10 µm^2^ and 80 µm^2^, respectively. The scale (microns per pixel) was configured using an image obtained with the UB203 microscope (UOP/Proiser R + D) with a 1× eyepiece, a 10× positive phase contrast objective and a Makler^®^ chamber, and photography using the concentration module of the CASA system, ISAS^®^ v1. The image was exported to the ImageJ software, and a horizontal line was drawn where the length between two lines of the Makler^®^ chamber grid to obtain the line length. Once this value was determined, the known value of 100 microns from the Makler^®^ chamber was divided by the length data to obtain the value of the scale to be used (microns/pixels). Afterward, parameters of boar semen were recorded for the concentration module of Open CASA v2, as well as the determined scale. Then, the videos with the trajectories and images previously collected by the CASA-Mot system were exported in AVI and JPG format, respectively, and uploaded to the Open CASA v2 software. Using this system as an extension complement to ImageJ, the variables of motility and sperm kinetics were determined, for which an adequate reference of the effective fields previously taken with the ISAS^®^ v1 system had to be made.

### 2.7. Assessment of Sperm Concentration with the Accuread^®^ Photometer

For the assessments of sperm concentration with a photometer, the Accuread^®^ equipment with a light source of 595 nm, optical fiber and LED (IMV Technologies, L’Aigle, France) was used. The photometer was previously calibrated with the diluent used for the ejaculates. For this, microcuvettes (specific for the Accuread^®^ photometer) were used. A dilution was made according to the recommendation in the equipment’s user manual (1:25, *v*:*v*), adding 2400 µL of diluent and 100 µL of ejaculate to the micro cuvettes. For each ejaculate, two replicates were examined, and the results of the concentration in M/mL (millions of sperm/milliliters of ejaculate) were recorded in a database with their respective references. A subsequent correction had to be made to this recorded data because the ejaculate sample was previously diluted at the farm with a 1:1 (*v*:*v*) ratio.

### 2.8. Statistical Analysis

Data collected from all assessments were subjected to exploratory analysis and tested for statistical assumptions such as normal distribution properties of sperm concentration using normal probability plots. This was done by calculating asymmetries and flattening coefficients for all analyses. The four methods used for assessments of sperm concentration were compared using a least squares regression line analysis. A Student’s *t*-test was used to compare sperm concentration methods. The results of the *t*-tests were systematically compared to a theoretical t-value with a 5% risk factor of accepted level of significance. The Student’s *t*-test and the straight-line equation were performed with IBM’s SPSS package for Windows (version 23.0, SPSS Inc., Chicago, IL, USA). Bland–Altman plots and Passing–Bablok methods of regression analyses for assessing the agreement between two measurement methods were done with XLSTAT statistical and data analysis solution version 2021.5.1. To determine the differences between measurement methods of total and progressive motility, an ANOVA was carried out with LSD Fisher’s test using the InfoStat package version 2020 (Centro de Transferencia InfoStat, FCA, Universidad Nacional de Córdoba, Argentina) [33].

Data resulting from ejaculate evaluations were analyzed with Bayesian statistics (this approach allows for a nuanced understanding of data, taking into account both the mean of the posterior distribution and the high posterior density or credibility interval, HPD). Additionally, the use of a guaranteed value k, which is the value of the interval [k, +∞] with a probability determined by the user, provides a measure of the difference between levels of treatment that is relevant to the assay. Although a difference between levels of treatment can be important, if HPD is high, the true value is typically irrelevant. The incorporation of prior knowledge into the analysis can be useful, accurate and informative. The guaranteed value k provides a threshold for the difference between levels of treatment that is considered to be meaningful. For a specific value of k, the difference between levels will have at least this value, with a probability determined by the user (for example, 80% or 95%) that can ensure that any differences between levels of treatment that are detected are of practical significance. We also explored the distribution properties of the data for all variables using histograms and normal probability plots to identify outliers or non-normal distributions. Sperm concentration was the response variable. For sperm concentration, a normal distribution with an identity link function was assumed. Other fixed factors that may have an impact on sperm concentration were also added to the model; these were the sire line, nested boar within the sire line, month in which semen was collected, and length of time between previous and present ejaculates. Additionally, a random residual effect was included in the model to account for the correlation between different ejaculates from the same boar, and the male number was included as a random effect. All analyses were carried out using Bayesian methodology that involves multiple variables with complex models. The posterior means of the differences between methods of measurement of sperm concentration (D) were estimated. The highest posterior density region at 95% (HPD_95%_) was calculated to estimate the range of values for a parameter that is most likely to contain the true value of that parameter or estimate the uncertainty associated. The probability of the difference being positive when D > 0 or negative when D < 0 (P_0_) were also calculated. Bounded uniform priors were employed for all effects. This is based on the assumption that the prior distribution for each effect is uniform over a certain range of values. Residuals were a priori normally distributed with mean 0 and variance σ^2^_e_. We considered one-third of the standard deviation (s.d.) of a trait to be a relevant value (R). This value was also used to determine the probability of relevance (P_R_), which is the probability of the difference being greater than R when D > 0 or lower than R when D < 0. The priors for variances were also assumed to be uniformly distributed within a certain range. The features of the marginal posterior distributions for all unknowns were estimated employing Gibbs sampling. Gibbs sampling is a Markov Chain Monte Carlo (MCMC) method used to estimate the posterior distribution of a parameter. Convergence was tested using Geweke’s Z criterion, which is a test to determine whether the MCMC chain has converged to the true posterior distribution [34]. Monte Carlo sampling errors were computed using the time series procedures [35]. The Rabbit software, which was developed by the Institute for Animal Science and Technology (Valencia, Spain), was used to determine differences between concentration measurement methods using a Bayesian approach.

## 3. Results

### 3.1. Overall Mean and Coefficient of Variation of the Posterior Distribution of the Sperm Concentration between Measure Methods and Genetic Sire Lines of Boars 

**Figure 1:**
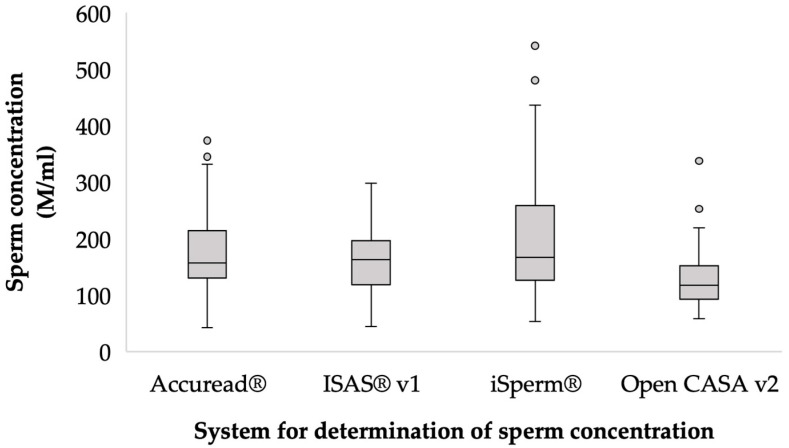
Sperm concentration (×10^6^·mL^−1^) according to estimation method. The area within the boxplot indicates 50% of the observations between the 25th and 75th percentiles, respectively. ——mean value; ┬ ┴—Minimum and maximum values within three standard deviation units (SD); data outside of confidence intervals (circles) are *outliers*.

The general means ± residual standard deviation (r.s.d.) of sperm concentration of ejaculate samples was 179.3 ± 76.6 M/mL. The overall coefficient of variation (CV) was 47.8%, with the highest posterior density region at 95% (HPD_95%_) between 154.7 and 204.7 M/mL (Figure 1).

The sperm concentration of genetic sire lines had CVs ranging from 49.5% to 50.8%, with mean values of 173.3 M/mL (Duroc × Pietrain) and 170.3 M/mL (Pietrain). The HPD_95%_ in the Duroc × Pietrain boars were 137.5, 212.2 M/mL, and in Pietrain boars (HPD_95%_) were 132.7, 207.5 M/mL. The probability of the differences between genetic sire lines Duroc × Pietrain and Pietrain were 0.53 and 0.52, respectively, indicating non-relevant differences in sperm concentration estimates between genetic sire lines (Table 1).

The measurement methods had significant differences (*p* < 0.05), with the iSperm^®^ values (193.8 M/mL) being the highest for concentration and Open CASA v2 (128.3 M/mL) having the lowest values.

**Figure 2:**
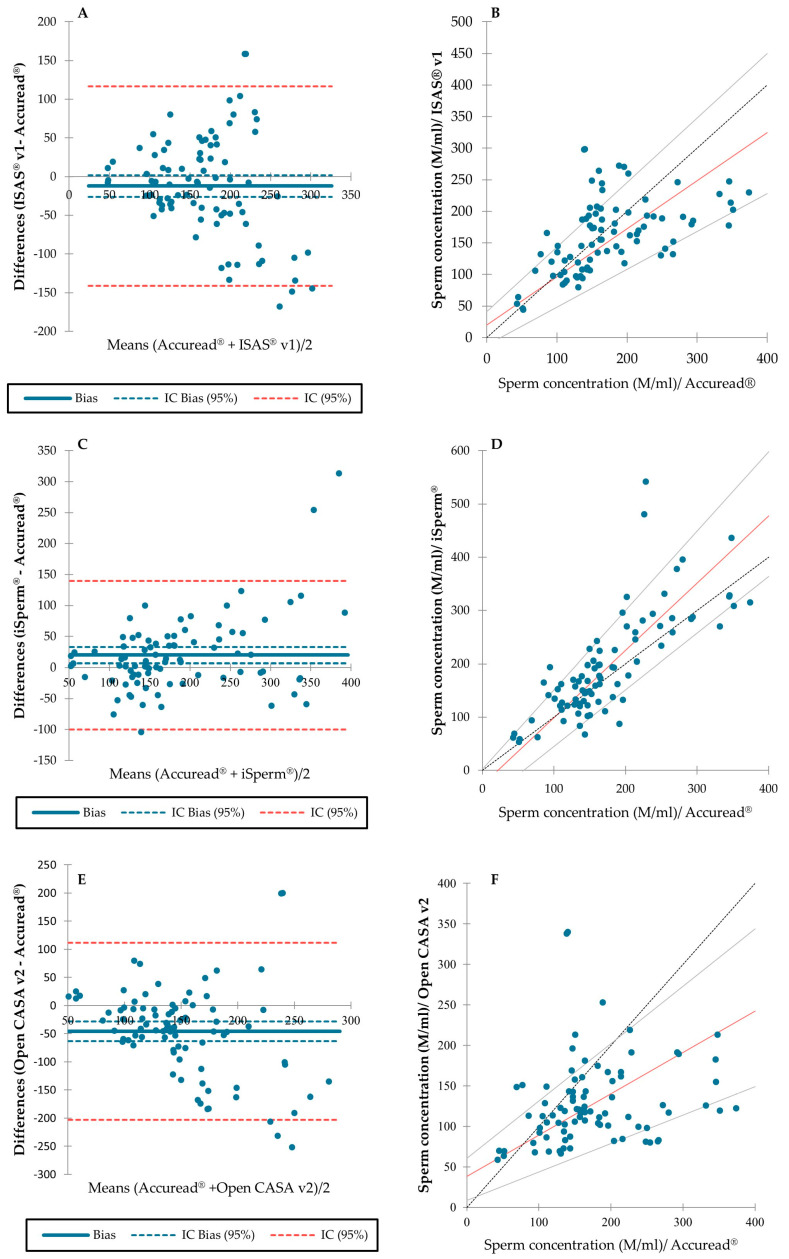
Bland–Altman plots and Passing–Bablok regressions for comparisons of sperm concentrations assessed using between different methods. (**A**) Bland–Altman plots for Accuread^®^ and ISAS^®^ v1. (**B**) Passing–Bablok regression for Accuread^®^ and ISAS^®^ v1. (**C**) Bland–Altman plots for Accuread^®^ and iSperm^®^. (**D**) Passing–Bablok regression for Accuread^®^ and iSperm^®^. (**E**) Bland–Altman plots for Accuread^®^ and Open CASA v2. (**F**) Passing–Bablok regression for Accuread^®^ and Open CASA v2.

Bland–Altman plots for ISAS^®^ v1 and Accuread^®^ showed a random pattern. The bias coefficient did not differ from 0 [95% confidence interval, CI: −26.404 to 1.816] (Figure 2A). There were three points beyond the bias limits (which were all above 140 M/mL). The Results of the Passing–Bablok regression revealed that the ISAS^®^ v1 analysis had an acceptable agreement with the Accuread^®^ analysis (slope [95% CI]: 0.761 to 1.022; intercept [95% CI]: −12.153 to 41.364); (Figure 2B).

Bland–Altman plots for iSperm^®^ and Accuread^®^ showed a random pattern, and the bias coefficient differed from 0 [95% CI: 6.647 to 32.843] (Figure 2C). There were two points beyond the bias limits (all above 250 M/mL). The results of the Passing–Bablok regression showed that the iSperm^®^ analysis reached an acceptable agreement with the Accuread^®^ outcome (slope [95% CI]: 1.063 to 1.490; intercept [95% CI]: −61.308 to 2.211); (Figure 2D).

Bland–Altman plots for Open CASA v2 and Accuread^®^ showed a random pattern; the bias coefficient differed from 0 [95% CI: −63.057 to −28.693] (Figure 2E). Four points were found beyond the bias limits (all above 200 M/mL). The results of the Passing–Bablok regression showed that the Open CASA v2 analysis had an acceptable agreement with Accuread^®^ analysis (slope [95% CI]: 0.351 to 0.708; intercept [95% CI]: 8.412 to 60.456); (Figure 2F). 

The relationships between sperm concentrations estimated with different methods, analyzed by least-square regressions, are shown in Supplementary Figure S1.

### 3.2. Sperm Progressive and Total Motility between Measure Methods

**Figure 3:**
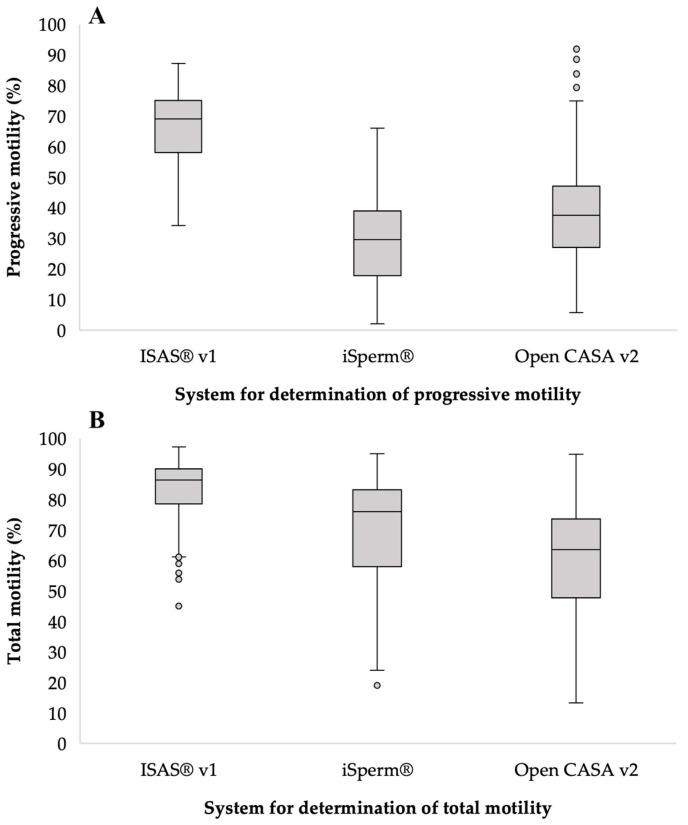
Progressive motility (**A**) and total motility (**B**) using different methods. Area within the boxplot indicates 50% of the observations between the 25th and 75th percentiles, respectively. ——mean value; ┬ ┴—Minimum and maximum values within three standard deviation units (SD); data outside of confidence intervals (circles) are *outliers*.

The general means ± standard deviation (SD) of sperm progressive motility and total motility of the ejaculate samples were 40.3 ± 17.2% and 63.1 ± 18.9%, respectively. The overall CV was 29.9% for progressive motility and 42.8% for total motility (Figure 3A and 3B, respectively). The differences between the Duroc × Pietrain and Pietrain sire lines were non-relevant (*p* > 0.05) in sperm progressive motility. On the other hand, the sperm total motility of the sire lines had values of 58.8% (Duroc × Pietrain) and 67.02% (Pietrain), and the differences were relevant (*p* < 0.05). 

The measurement methods had significant differences (*p* < 0.05), with the ISAS^®^ v1 values (82.1 ± 12.2%) being the highest for total motility and Open CASA v2 (59.9 ± 17.8%) having the lowest values. 

**Figure 4:**
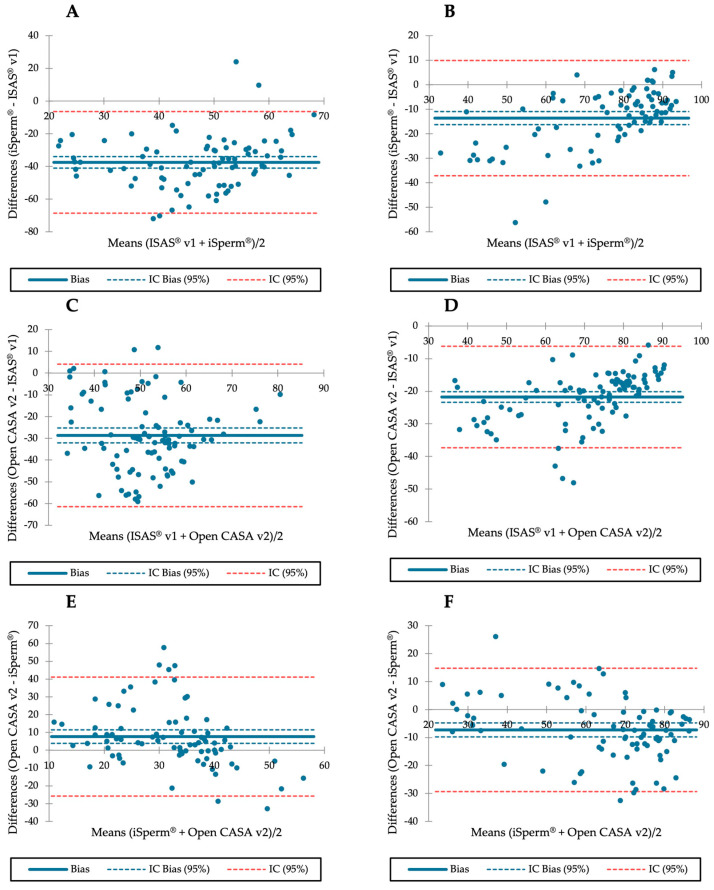
Bland–Altman plots comparisons of progressive and total sperm motility assessed using different methods. (**A**) Bland–Altman plots for progressive motility between ISAS^®^ v1 and iSperm^®^. (**B**) Bland–Altman plots for total motility between ISAS^®^ v1 and iSperm^®^. (**C**) Bland–Altman plots for progressive motility between ISAS^®^ v1 and Open CASA v2. (**D**) Bland–Altman plots for total motility between ISAS^®^ v1 and Open CASA v2. (**E**) Bland–Altman plots for progressive motility between iSperm^®^ v1 and Open CASA v2. (**F**) Bland–Altman plots for total motility between iSperm^®^ v1 and Open CASA v2.

The Bland–Altman plots for iSperm^®^ and ISAS^®^ v1 exhibited a random pattern of progressive motility; the bias coefficient did not differ with 0 [95% CI: −41.047 to −33.988] (Figure 4A). Nonetheless, there were two points beyond the bias limits (all above 9%). For total motility, the bias coefficient did not differ from 0 [95% CI: −16.295 to −10.960] (Figure 4B). However, there were two points beyond the bias limits (all below −47%).

The comparison of Open CASA v2 and ISAS^®^ v1 using Bland–Altman plots showed a random pattern of progressive motility, and the bias coefficient did not differ with 0 [95% CI: −32.101 to −25.253] (Figure 4C). For total motility, the bias coefficient neither differs with 0 [95% CI: −23.391 to −20.140] (Figure 4D). However, there were three points beyond the bias limits (which were all below −42%). 

The differences in progressive motility between Open CASA v2 and iSperm^®^ using the Bland–Altman plots showed a random pattern, and the bias coefficient did not differ with 0 [95% CI: 3.870 to 11.463] (Figure 4E). However, there were four points beyond the bias limits (which were all above 31%). For total motility, the bias coefficient neither differs with 0 [95% CI: −9.782 to −4.773] (Figure 4F). However, there was one point beyond the bias limits (which was below −32%).

Passing–Bablok regressions for sperm progressive motility and for total sperm motility, estimated using different methods, are shown in Supplementary Figure S2.

### 3.3. Mean and Highest Posterior Distribution Interval at 95% of the Sperm Concentration between Measure Methods

Mean values of sperm concentration with Accuread^®^, ISAS^®^ v1, iSperm^®^ and Open CASA v2 were 174.0, 161.5, 193.8 and 128.3 M/mL, respectively, with the lowest density interval at 95% of 93.5 (Open CASA v2 = 99.3, 155.9 M/mL) and the highest interval HPD_95%_ of 224.2 M/mL (iSperm^®^ = 167.0, 224.2), with the probability of the differences between methods being 0.61–0.99. The guaranteed value (k_80_) (which is defined to determine the range of values that are likely to be accurate with a probability of 80%) showed that the estimated values of sperm concentration were closer to mean values in the different methods used to assess sperm concentration (Table 2). 

### 3.4. Bayesian Contrast Analysis of Relevance between Sire Genetic Line and Sperm Concentration Methods

Differences in both genetic sire lines for sperm concentration were non-relevant, with a probability of differences of 0.55 and the probability of relevance of 0.14 (Table 3). Differences in four methods to measure sperm concentration were relevant, with a probability of differences between 0.86 and 1.00. The sperm concentration values in Accuread^®^ system, iSperm^®^ and Open CASA v2, (Accuread^®^—Open CASA v2, mean of the marginal posterior distribution of the difference (D) = 45.7 M/mL; iSperm^®^—Open CASA v2, D = 65.4 M/mL) was the greater differences between methods with a probability of differences of 1.00 in both cases. Relevant intermediate differences were found in the systems ISAS^®^ v1 with regard to iSperm^®^ and Open CASA v2 (ISAS^®^ v1—iSperm^®^, D = −32.3 M/mL; ISAS^®^ v1—Open CASA v2, D = 33.1 M/mL). The methods that show the lowest differences were Accuread^®^ with regard to ISAS^®^ and iSperm^®^ (Accuread^®^—ISAS^®^ v1, D = 12.6 M/mL; Accuread^®^—iSperm^®^, D = −19.7 M/mL) with a lower probability of difference although relevant (Table 3).

### 3.5. Seasonal Effect on Sperm Concentration in Boar Ejaculates

There was a seasonal effect on sperm concentration. There was a lower sperm concentration during the months of May (mean of 167.3 M/mL and k80 of 156.8 (HPD_95%_ = 141.9, 191.6 M/mL)) and June (mean of 133.1 M/mL and k80 of 125.4 (HPD_95%_ = 116.1, 152.8 M/mL)). The highest sperm concentration was observed from August–October, with an estimated k80 of 219.1 M/mL in September. There were relevant differences in sperm concentration between months of ejaculate collection (Table 4).

## 4. Discussion

In this comparative study, the results revealed differences between the techniques, even though some of these differences were non-relevant. We estimated the probability of the contrast being higher than a value considered to be relevant from biological or economic points of view. The value k ensures that the difference between levels will have at least this value with a given probability (e.g., 80% or 95%). Thus, the k80 value could provide an effective tool for improving the accuracy of sperm concentration. The Bayesian approach, as used in our study, allows us to determine the probability of an interval of values within which the true value lies, with a predetermined level of probability based on certain prior knowledge. Using a 95% Bayesian probability interval thus means there is a 95% probability that the interval contains the unknown true value.

Accurate estimation of sperm concentration is essential for determining the optimal semen doses and predicting the likelihood of successful artificial insemination and fertilization. Data analysis of methods for the estimation of sperm concentration were consistent because the guaranteed value was closer than the sperm concentration mean. Previous studies that compared the accuracy of sperm concentration using three methods, hemocytometer, spectrophotometer and flow cytometry, found that when defining threshold values, the k80 value could be closer to the mean values in all three methods, indicating that the use of this measure can improve the accuracy of sperm concentration estimation across different methods [36,37,38].

Our results showed that sperm motility and concentration estimates have relevant differences between methods. These could be explained by the method of estimation [39], calibrations of systems [3,10], settings of systems and algorithms [40,41], the luminous capacity of systems [42], or the type of chamber used for analysis [43,44,45,46,47]. Male differences could also be a source of variation. However, in this study, there were no relevant differences in sperm concentration estimates among sire lines. Pietrain and Duroc × Pietrain boars had estimated sperm concentrations in the ejaculate of around 170 M/mL, with a guaranteed value at 80% of 160 M/mL.

ANOVAs revealed relevant differences among the three methods of motility estimation, with the ISAS^®^ v1 presenting greater values compared with iSperm^®^ and Open CASA v2, the latter showing the lowest values for total and progressive motility. Earlier studies also revealed similar differences in other species (stallion [20,28,42]; fish [22]) with these systems. In this case, variation could be due to the temperature of analysis [10,48], presence of agglutination [49], or composition of the ejaculates and their heterogeneity, and can be explained by the existence of different cell groupings or subpopulations within ejaculate [50,51]. 

A lower (or higher) value of a sperm parameter in normal conditions could be related to the subestimation of the method. For this reason, the assessment of sperm concentration by a Bayesian approach is useful as it incorporates, first, prior knowledge about the biology of sperm concentration and, second, the variability of CASA measurements to examine the biological relevance of these measures estimated by using CASA methods. This is important because concentration may be used as a selection parameter in farms [1,6,52,53] Both the underestimation [14] and overestimation [18,54] could affect the economic yield of farms.

The Bayesian contrast analysis indicated that sperm concentrations measured with Accuread^®^ were higher than those from the Open CASA method and that these differences were relevant. These results resemble those in another study that compared a CASA system and two spectrophotometers [7]. Moreover, the Bayesian contrast analysis showed that in a comparison of Accuread^®^ with iSperm^®^ and ISAS^®^ v1 systems, differences with the latter one were not relevant. When comparisons were made between CASA methods such as iSperm^®^, ISAS^®^ v1 and Open CASA, the contrast of differences in sperm concentration estimates was statistically relevant. Comparison between a manual system such as Accuread^®^ and CASA systems analysis have shown significant variations in the past [9], which were explained by technical errors. In our study, we controlled most of the error factors that could introduce variation in the models. Another study revealed values of correlation for concentration (R^2^ = 0.95) with the SCA CASA system that was higher than that observed in our work. The other study involved the analysis of 287 semen samples using a CASA prototype with customized software. The path-tracking algorithm used in the software allowed for the accurate tracking of sperm movement, which is necessary for determining sperm concentration [40]. The authors reported a high rate of convergence, significant for concentration determination (R^2^ = 0.94; *p* < 0.001 on 287 samples), indicating that the results obtained were consistent and reliable. The use of customized software in the CASA systems allows for more accurate measurements and reduces the potential for human error and inconsistencies in the results.

The present study provides valuable tools for analyses of semen evaluation systems, which can be used in other species or under different conditions, as variations in protocols can lead to inconsistent results. The sperm analysis systems are validated, and protocols are standardized to improve the accuracy of sperm concentration estimates. Altogether, this would result in better knowledge of methods used to estimate parameters of semen quality [55,56,57,58] and represent new opportunities for producers that can access new technologies [23,28,29,59,60] with greater objectivity [61,62,63] that could complement or replace methods for manual or visual analyses of semen quality, and this could help them make more informed decisions about breeding and reproduction [16,64,65]. Further research is nonetheless needed to refine the estimation methods of boar sperm concentration and motility and to determine the most appropriate method for different applications and animal production systems and conditions. 

## 5. Conclusions

This study revealed relevant differences between methods used for the estimation of boar sperm concentration and motility. The data show that the iSperm^®^ presented greater sperm concentration values, while Open CASA v2 yielded lower values. For total and progressive motility, the ISAS^®^ v1 returned greater values, while Open CASA v2 showed lower values in both variables. Differences in estimation methods of boar sperm concentration and motility by CASA systems suggest the need for improved refinement.

## Figures and Tables

**Table 1:** The mean value, high posterior density interval, and guaranteed value for sperm concentration in two genetic sire lines of boars.

	Mean	HPD_95%_^1^	P_0_^2^	GV*_k_* ^3^
Duroc × Pietrain	173.3	137.5, 212.2	0.53	159.0
Pietrain	170.3	132.7, 207.5	0.52	156.3

^1^ HPD_95%_—highest posterior density region at 95%; ^2^ P_0_—probability of the difference being greater than zero when D > 0 and probability of the difference being lower than zero when D < 0; ^3^ GV*_k_*—guaranteed value (k = 0.80). Number of boars: 10.

**Table 2:** Mean value, high posterior density interval, and guaranteed value for boar sperm concentration in four measure methods.

	Mean	HPD_95%_^1^	P_0_^2^	GV*_k_* ^3^
Accuread^®^	174.0	147.5, 204.0	0.61	162.5
ISAS^®^ v1	161.5	132.9, 190.4	0.83	149.8
iSperm^®^	193.8	167.0, 224.2	0.78	182.3
Open CASA v2	128.3	99.3, 155.9	0.99	116.8

^1^ HPD_95%_—highest posterior density region at 95%; ^2^ P_0_—probability of the difference being greater than zero when D > 0 and probability of the difference being lower than zero when D < 0; ^3^ GV*_k_*—guaranteed value (k = 0.80). Number of boars: 10.

**Table 3:** Boar sperm concentration and estimated marginal posterior distribution of differences between two sire genetic lines and four measure methods.

	D ^1^	HPD_95%_^2^	P_0_^3^	P_R_^4^	P_S_^5^	GV*_k_* ^6^
Boar breed effect						
Duroc × Pietrain—Pietrain	2.96	−50.3, 54.0	0.55	0.14	0.76	−16.3
Sperm concentration measure methods effect						
Accuread^®^—ISAS^®^ v1	12.6	−10.8, 35.8	0.86	0.08	0.92	2.9
Accuread^®^—iSperm^®^	−19.7	−41.8, 3.0	0.96	0.21	0.79	−9.8
Accuread^®^—Open CASA v2	45.7	22.2, 67.8	1.00	0.93	0.07	36.3
ISAS^®^ v1—iSperm^®^	−32.3	−54.7, −9.9	1.00	0.61	0.39	−22.4
ISAS^®^ v1—Open CASA v2	33.1	11.9, 56.9	1.00	0.65	0.35	23.4
iSperm^®^—Open CASA v2	65.4	42.1, 87.8	1.00	1.00	0.00	55.5

^1^ D—mean of the marginal posterior distribution of the difference between Duroc × Pietrain and Pietrain, and Accuread^®^, ISAS^®^ v1, iSperm^®^, and Open CASA v2. ^2^ HPD_95%_—highest posterior density region at 95%; ^3^ P_0_—probability of the difference being greater than zero when D > 0 and probability of the difference being lower than zero when D < 0; ^4^ P_R_—probability of relevance (relevant value: R), it is the probability of the difference being greater than R when D > 0 and less than R when D < 0. ^5^ P_S_—probability of similitude, it is the probability of the absolute value of a contrast being lower than a relevant value. ^6^ GV*_k_*—guaranteed value (k = 0.80). Number of boars = 10.

**Table 4:** Mean value, high posterior density interval, and guaranteed value for boar sperm concentration at different months.

Month	Mean	HPD_95%_^1^	P_0_^2^	GV*_k_* ^3^
May	167.3	141.9, 191.6	0.88	156.8
June	133.1	116.1, 152.8	1.00	125.4
July	186.2	160.8, 211.1	0.53	175.4
August	191.1	169.3, 213.4	0.70	182.0
September	232.1	204.4, 263.0	1.00	219.1
October	198.7	162.7, 233.9	0.77	183.3

^1^ HPD_95%_—highest posterior density region at 95%; ^2^ P_0_—probability of the difference being greater than zero when D > 0 and probability of the difference being lower than zero when D < 0; ^3^ GV*_k_*—guaranteed value (k = 0.80). Number of boars: 10.

## Data Availability

The data presented in this study are available within the article and/or its Supplementary Materials.

## Supplementary Materials

The following supporting information can be downloaded at: https://www.mdpi.com/article/10.3390/ani13101622/s1, Figure S1. Relationships between sperm concentrations assessed with different methods. (A) Regression between concentration values estimated with Accuread^®^ and ISAS^®^ v1. Least-square regression line (*n* = 50). Model: *y* = 0.76*x* + 20.17. (B) Regression between concentrations estimated with Accuread^®^ and iSperm^®^. Least-square regression line (*n* = 50). Model: *y* = 1.26*x* − 27.28. (C) Regression between concentrations estimated with Accuread^®^ Open CASA v2 methods. Least-square regression line (*n* = 50). Model: *y* = 0.51*x* + 37.97. Figure S2. Passing–Bablok regressions for sperm progressive motility and total sperm motility estimated using different methods. (A) Regression for sperm progressive motility between ISAS^®^ v1 and iSperm^®^. (B) Regression for sperm total motility between ISAS^®^ v1 and iSperm^®^. (C) Regression for sperm progressive motility between ISAS^®^ v1 and Open CASA v2. (D) Regression for sperm total motility between ISAS^®^ v1 and Open CASA v2. (E) Regression for sperm progressive motility between iSperm^®^ and Open CASA v2. (F) Regression for sperm total motility between iSperm^®^ and Open CASA v2.
