# Supplementary material for: Are There Differences between Methods Used for the Objective Estimation of Boar Sperm Concentration and Motility?
Source: Animals (Basel). 2023 May 12;13(10):1622. doi: 10.3390/ani13101622 (PMC10215348; doi:10.3390/ani13101622)
Supplement: Supplementary file 1 [file animals-13-01622-s001.zip › animals-2313438-supplementary.pdf]

## SUPPLEMENTARY MATERIALS

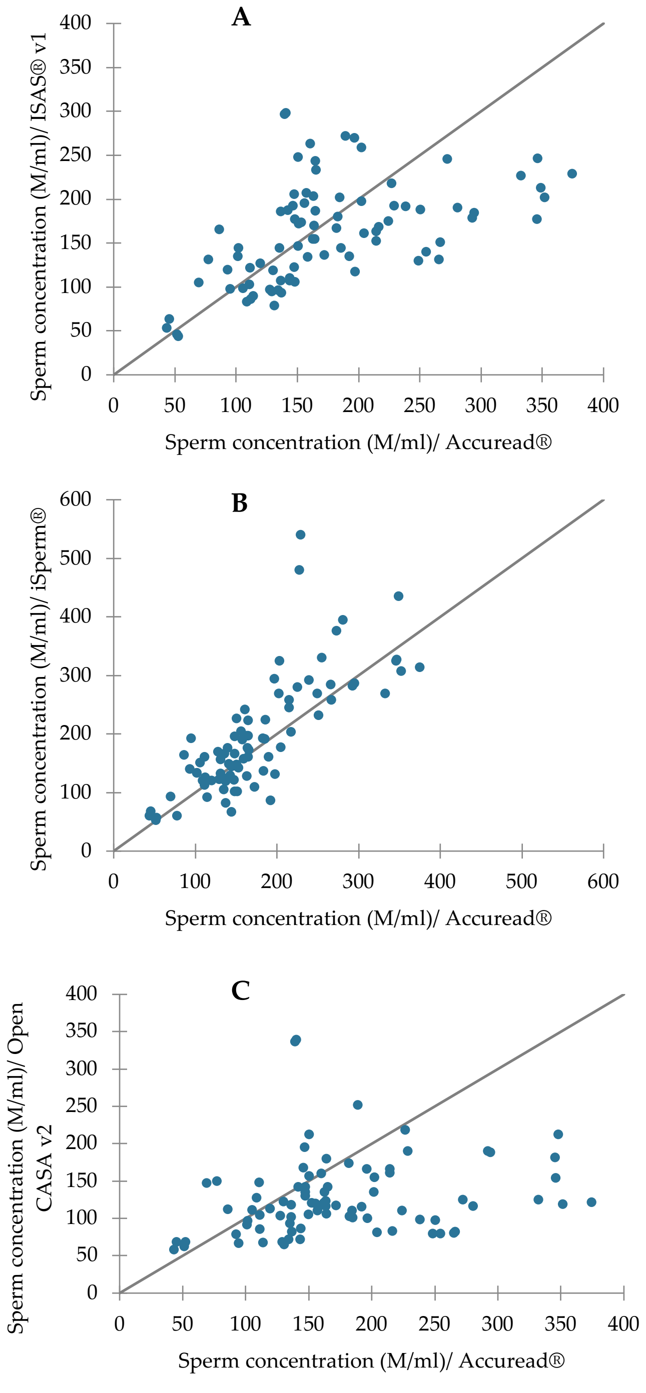


**Figure S1.** Relationships between sperm concentrations estimated with different methods. **(A)** Regression between concentration values estimated with Accuread® and ISAS® v1. Least-square regression line (n=50). Model: y = 0.76x + 20.17. **(B)** Regression between concentrations estimated with Accuread® and iSperm®. Least-square regression line (n=50). Model: y = 1.26x - 27.28. **(C)** Regression between concentrations estimated with Accuread® OpenCASA® methods. Least-square regression line (n=50). Model: y = 0.51x + 37.97.

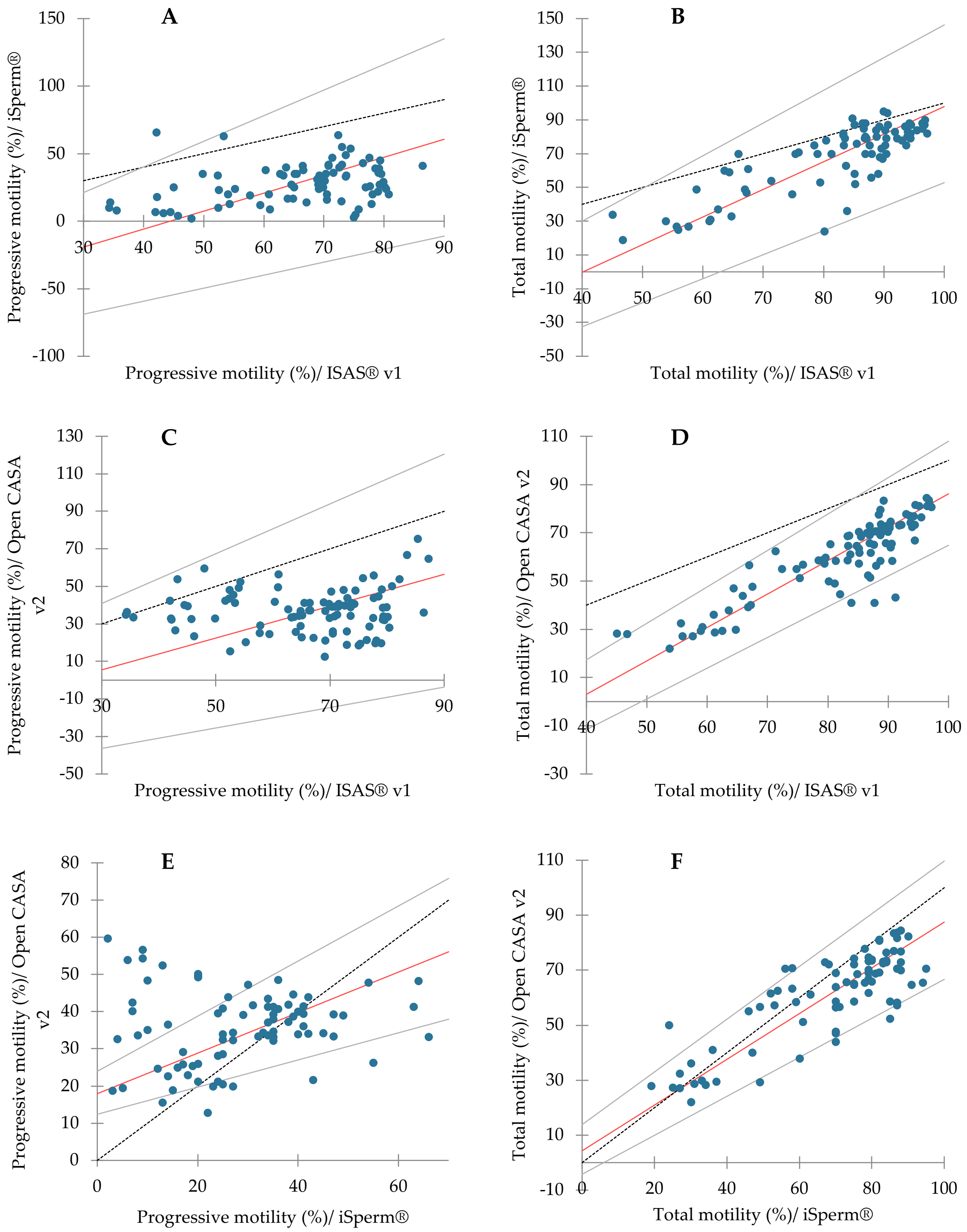


**Figure S2.** Passing-Bablok regressions for sperm progressive motility and total sperm motility estimated using different methods. **(A)** Regression for sperm progressive motility between ISAS® v1 and iSperm®. **(B)** Regression for sperm total motility between ISAS® v1 and iSperm®. **(C)** Regression for sperm progressive motility between ISAS® v1 and Open CASA v2. **(D)** Regression for sperm total motility between ISAS® v1 and Open CASA v2. **(E)** Regression for sperm progressive motility between iSperm® and Open CASA v2. **(F)** Regression for sperm total motility between iSperm® and Open CASA v2.
